# Integrating the dual-system theory in motor: dynamic framework construction for motor decision-making

**DOI:** 10.3389/fpsyg.2025.1720922

**Published:** 2026-01-13

**Authors:** Zidong Huang, Xiaozhuo Wei, Xutao Liu, Yechen Yang

**Affiliations:** 1 School of Physical Education, Jiangsu University of Science and Technology, Zhenjiang, China; 2 School of Management, Shanghai University, Shanghai, China

**Keywords:** motor decision-making, dual-system theory, task constraints, physiological state, experience level

## Abstract

**Introduction:**

Existing research on motor decision-making from a dual-system perspective is often limited by static and dichotomous approaches, failing to fully explain the dynamic fluctuations in decision performance in real sports contexts. Most studies focus on isolated factors, lacking a systematic integration of the interactive effects among external tasks, internal states, and experience levels. Therefore, this study aims to construct a theoretical framework that integrates these three dimensions to reveal the dynamic interaction mechanisms of the dual systems in motor decision-making.

**Methods:**

Based on a comprehensive literature review and critical analysis, this theoretical article proposes a framework centered on three core dimensions:task constraints (time pressure, task complexity), physiological states (fatigue, arousal), and experience level (expert-novice paradigm). It further clarifies the specific pathways through which these dimensions influence the dual-system interaction by modulating cognitive resources.

**Results:**

The study yields a coherent theoretical framework that systematically outlines how the three dimensions interact to influence the dynamic interaction of dual systems. This framework provides a mechanistic explanation for phenomena such as athletes’ performance fluctuations and decision-making errors, confirming the proposed integrative perspective.

**Discussion:**

The proposed framework not only offers a more systematic and ecologically valid explanatory model for the field of motor decision-making but also, as the theoretical foundation for a series of subsequent studies, provides top-level design and methodological guidance for future empirical research. It promotes a shift from isolated factor analysis to a multi-dimensional interactive view, opening perspectives for exploring complex decision-making mechanisms in real-world sports environments.

## 1 Introduction

In the realm of competitive sports, athletes are required to rapidly assess and interpret the competitive environment, enabling them to make informed decisions and subsequently select and implement the most efficacious technical actions (Tenenbaum and Filho, 2017). Elite athletes exhibit a dual nature in decision-making: they are capable of executing exceptional actions intuitively, yet they can also make instinctive errors during critical moments. This phenomenon highlights a fundamental issue within the human cognitive system: how to achieve a dynamic balance between automatic intuition and rational control under conditions of fluctuating constraints.

Such problems are structured in the two-system theory of motor cognition (Furley et al., 2015). The central theory posits that human cognitive processing relies on two functionally distinct yet interacting information-processing systems: intuitive system(System 1), and analytical system(System 2). In complex sports performance, these systems should be seen as interacting processes on a continuum rather than separate entities. Our framework examines how their dynamic interaction and changing dominance are influenced by contextual and individual factors. This theoretical framework has been further elucidated and expanded across various dimensions. Information processing theory emphasizes the critical importance of conscious analysis, rule application, and the regulation of working memory. It advocates for reliance on deliberate cognitive calculations to achieve optimal solutions in complex scenarios (Eggen, 2020; Fitriani et al., 2025). Furthermore, this theory illustrates the plasticity inherent in cognitive control (Kanaev, 2023; Dere, 2024). Neurobehavioral evidence contributes to the ongoing debate, as functional magnetic resonance imaging (fMRI) and electroencephalogram (EEG) studies reveal that intuitive decisions are linked to the rapid activation of the basal ganglia (BG) and amygdala (Amy), corresponding to System 1 processing (Roca and Williams, 2017; Kwon et al., 2019; Chen et al., 2024; Seidel-Marzi et al., 2024), analytical decisions engage the prefrontal cortex (PFC) and elicit P300 components, indicative of analytical system processing (Chen et al., 2024).

However, the dual-system theory is not without its challengers. The ecological dynamics framework (Araújo et al., 2025), grounded in Gibsonian realism, it advocates a non-dualistic view in which behavior emerges from a continuous perception-action cycle, contrasting fundamentally with the dual-system theory’s emphasis on internal cognitive dynamics. This divergence is reflected in distinct units of analysis: while ecological dynamics examines the athlete-environment system as a whole, the dual-system theory focuses on individual-level psychological mechanisms. The decision to employ the dual-system approach in this study is a pragmatic one, driven by our specific research objective to develop a model that is both computable and empirically verifiable. While ecological dynamics is adept at providing a holistic explanation for the emergence of behavior, the dual-system framework offers an operationalizable structure for quantifying the dynamic trade-offs between cognitive modes. It provides a lexicon of constructs, such as intuitive versus analytical processing, which can be associated with manipulable experimental variables, such as time pressure, and measurable neurophysiological indicators, such as P300 amplitude and PFC activity. This capacity for quantification is crucial for achieving our goal of transitioning from theoretical description to predictive modeling. Therefore, we utilize the dual-system theory not as a definitive explanation of cognition, but rather as a practical instrument (Furley et al., 2015). Its utility is found in offering a testable heuristic that enables the measurement of the “how” and “when” of processing trade-offs. This focus is robustly supported by empirical evidence demonstrating a duality in sports contexts, exemplified by the advantage of intuition under time constraints (Roca and Williams, 2017; Tong and Li, 2021; Lucia et al., 2023).

While dual-system theory offers a comprehensive framework through the interaction, it encounters several limitations when applied to real-world motion scenarios. Firstly, research on this correlation predominantly relies on static experimental paradigms, such as temporal blocking and spatial masking, which overlook the dynamic transformation characteristics of the dual-system in real contexts. Secondly, the utilization of physiological indicators within dual-system theory remains inadequate. Current studies often examine physiological indices in isolation and fail to elucidate how these indicators relate to the interaction mechanism of the two systems comprehensively. Thirdly, there is a lack of integration of individual stable experiential traits with real-time tasks and physiological factors, which neglects the fundamental adjustment of experience level within the two-system model. Fourthly, theoretical validation methods are lagging, with a deficiency in computational models and data analysis techniques capable of accurately quantifying the dynamic interaction processes of the two systems. Collectively, these limitations hinder the ability to effectively describe the intricate nature of decision-making in real motion contexts.

In light of these considerations, this paper posits that these limitations underscore a substantial research gap in the empirical application of the dual system paradigm, rather than suggesting its total ineffectiveness. Building upon this foundation, the present paper seeks to propose a comprehensive three-dimensional theoretical framework that integrates task constraints, physiological state, and experience level. It endeavors to elucidate the dynamic interaction mechanisms between dual systems in motor decision-making and to clarify the operational pathways of each dimension. These three dimensions were chosen because they represent the fundamental factors influencing decision-making: the immediate external environment (task constraints), the individual’s internal resource state (physiological state), and the enduring cognitive structures that develop over time (experience level). It moves beyond asking which system is active, to modeling how their dynamic trade-off is co-determined by the confluence of task, physiology, and experience. This effort aims to establish a theoretical basis for the development of a computable and empirically verifiable model. Our integrated framework models the interplay of task constraints, physiological states, and experiential factors in shaping the decision-making process. This approach not only addresses the limitations inherent in static dual-system models but also integrates a dynamic, context-sensitive perspective that aligns with the emphasis on perception-action coupling within ecological dynamics. Furthermore, the paper aspires to enhance the understanding of the cognitive mechanisms underlying exercise and to offer hypothesis-driven and methodological support for future quantitative research. Ultimately, this work aims to facilitate the transition from theoretical description to predictive modeling in the domain of exercise decision-making research.

## 2 Theoretical basis

### 2.1 Dual-system theory

The dual-system theory provides an influential, though debated, conceptual framework in cognitive psychology. It posits that decision-making can be usefully described as involving two interacting modes of processing: the rapid, intuitive System 1 and the slower, analytical System 2 (Gulati et al., 2021). While this theory remains subject to debate—particularly given challenges from ecological dynamics and predictive processing frameworks that reject strict binary classifications—it serves as a valuable analytical tool in the study of motor decision-making. Its utility lies in facilitating the quantification of the dynamic trade-off between fast-intuitive and slow-deliberative processes. Crucially, it conceptualizes these two modes as complementary endpoints on a continuum of cognitive control, rather than as strictly distinct entities, thereby providing a lexicon for understanding fluctuations in decision-making performance.

The intuitive system operates rapidly and automatically, heavily reliant on emotional processing and heuristic-based pattern recognition. It is frequently engaged in tasks such as the swift recognition of facial expressions or immediate reactions to sudden auditory stimuli (Aven, 2018; Maxwell et al., 2021; Robins, 2022). In contrast, the analytical system operates at a slower pace, necessitates conscious engagement and oversight, and is chiefly accountable for logical reasoning, critical analysis, and intricate computational tasks (Zhou and Pitt, 2024). Research indicates that intuitive system produces preliminary judgments, which analytical system subsequently intervenes to evaluate or modify. However, intuitive system often prevails in situations where cognitive resources are limited, time constraints are present, or motivation is insufficient (Da Silva, 2023; Xu et al., 2025). A comparison of the core characteristics of the two is shown in Table 1.

**Table 1:** Comparison of core characteristics of two systems.

Feature	Intuitive system	Analytical system
Speed	Fast, Automatic	Slow, Effortful
Process	Unconscious	Conscious
Method	Relies on Emotion, Heuristics	Relies on Logic, Rules
Energy consumption	Low Cognitive Load	High Cognitive Load
Role	Governs daily, quick decisions	Responsible for complex reasoning and correcting intuition

Despite the valuable insights provided by alternative frameworks such as ecological dynamics, the dual-system theory remains essential for a comprehensive understanding of motor decision-making. This is because it specifically addresses a fundamental and irreducible aspect of the athletic experience: the conscious tension between deliberate strategy and impulsive action. While ecological dynamics effectively describes the emergent, coupled behavior of the athlete-environment system, it does not fully account for the internal, subjective conflict reported by athletes between “overthinking” (over-reliance on the analytical system) and “playing on instinct” (dominance of the intuitive system). This phenomenology of intra-psychic conflict is particularly pronounced under high-pressure conditions, where time for deliberate perception-action coupling is limited. The dual-system theory is uniquely capable of modeling this internal conflict and the dynamic trade-offs that characterize expert performance, thereby underscoring its necessity. Our framework employs dual-system theory not merely for its convenience, but because it is inherently aligned with the phenomenon under investigation.

### 2.2 Application and evidence of dual-system theory in motor decision research

In the field of motor decision-making research, the two-system theory offers a robust theoretical framework for comprehending the cognitive processing mechanisms employed by athletes, and it has garnered substantial empirical support. Building on the aforementioned summary of intuitive system, this theory can be articulated as the pattern recognition and implicit knowledge developed by athletes through extensive training in sports contexts, enabling them to make rapid decisions. For instance, elite football players are capable of assessing the positions of defenders and determining whether to shoot or pass immediately upon receiving the ball (Seidel-Marzi et al., 2024). While intuitive decision-making is often highly efficient, it is susceptible to cognitive biases (Garces-Velastegui, 2024). Conversely, analytical system is integral to strategic planning (Wei et al., 2022). For instance, coaches must systematically analyze the characteristics of opponents and devise appropriate strategies (Zhang, 2024). Similarly, athletes engage analytical system for self-reflection and technical enhancement (Wang, 2022). Furthermore, decisions in sports often involve balancing risks and benefits (Cui et al., 2022). For example, climbers must assess weather conditions and their physical state to determine whether to proceed with their ascent (Pennock, 2020). Although the analytical capabilities of analytical system facilitate a more comprehensive risk assessment and rational decision-making, they are constrained by the available time and cognitive resources (Seidel-Marzi et al., 2024).

Numerous empirical studies have demonstrated that athletes’ skill levels substantially influence the utilization of both cognitive systems. Elite athletes, due to their extensive experience and advanced pattern recognition abilities, are more adept at employing intuitive system for efficient and accurate decision-making (Seidel-Marzi et al., 2024). In contrast, novice athletes tend to depend more heavily on analytical system for deliberate analysis, which consequently leads to slower decision-making processes (Seidel-Marzi et al., 2024). Nevertheless, the distinction between expert and novice should not be oversimplified. It is important to recognize that an expert’s rapid decisions, although seemingly intuitive, are underpinned by a strategic awareness and pattern recognition honed through extensive experience. This complexity challenges the straightforward dichotomy between speed and cognitive effort. Situational factors play a crucial role in decision-making processes, particularly within highly complex and uncertain competitive environments. Athletes are often required to make decisions under rapidly changing conditions, which places an increased cognitive load on intuitive system. Concurrently, the constraint of limited time hinders the ability to conduct detailed analyses associated with analytical system (Seidel-Marzi et al., 2024). Under conditions of time pressure, individuals are more inclined to rely on the swift, intuitive processes associated with intuitive system, as opposed to the more deliberate and analytical processes characteristic of analytical system (Garces-Velastegui, 2024). The physiological state of athletes plays a crucial role in modulating the functioning of both cognitive systems. Physical fatigue can deplete cognitive resources, thereby impairing the regulatory function of analytical system. This impairment leads to a greater dependence on the intuitive responses of intuitive system, consequently elevating the risk of decision-making errors (Ahuja, 2024). While the optimal arousal level will help improve the processing efficiency of intuitive system and the concentration of analytical system, and promote the synergy between them (Seidel-Marzi et al., 2024). An increase in cognitive load may compel athletes to rely on the more resource-efficient intuitive system, potentially impacting athletic performance during multitasking situations. This shift reflects conflicts in the allocation of cognitive resources across various tasks. Furthermore, extended training periods can enhance the level of automation and facilitate the dynamic equilibrium and optimization between the two systems. This assertion is corroborated by research on the interaction mechanisms of memory traces in neurobiology (Grossberg, 2020).

In recent years, with the continuous development of cognitive neuroscience and artificial intelligence (AI) technology (Bhatia, 2021; Derosiere, 2023),the research methods of motor decision making are constantly enriched, such as Brain-Computer Interface technology, which provides a new way to analyze the neural mechanism of decision making in real time (Zeng et al., 2023). In addition, the proposed multi-system cognitive model will also provide a new research perspective for the study of motor decision-making (Boag et al., 2021; Dere, 2024).

### 2.3 Review of the research

As previously examined, although the dual-system theory provides a comprehensive meta-framework for elucidating motor decision-making, and existing research has successfully validated its fundamental principles, certain overarching limitations persist within this field. These limitations impede a deeper understanding of the complex mechanisms underlying motor decision-making.

Firstly, contemporary research primarily investigates individual influencing factors in isolation, thereby lacking a comprehensive theoretical framework. Numerous studies have examined the isolated effects of variables such as skill level, time pressure, and cognitive load. However, in real-world sports contexts, these factors do not function independently; instead, they coexist and interact in complex ways. For example, the decision-making process of a fatigued novice athlete faced with complex tactical decisions at the conclusion of a competition is simultaneously influenced by multiple factors. Current theoretical models face challenges in clearly articulating how these factors dynamically interact to influence the balance between dual-systems thinking. Therefore, it is imperative for future research to formulate a theoretical framework that systematically and comprehensively incorporates multiple influencing factors.

Second, there continues to be an insufficient and indirect emphasis on critical dimensions, particularly concerning physiological states. As delineated in Section 1.2, physiological factors, including fatigue and arousal, play a pivotal role in modulating the functionality of the two systems. Nevertheless, a substantial body of behavioral research has predominantly regarded these factors as potential confounding variables or has evaluated them through self-reported measures, rather than directly and objectively quantifying their impacts. This methodological limitation impedes a precise understanding of the ways in which physiological states affect the interaction mechanisms of the two systems, whether by depleting cognitive resources or modifying attention allocation. Therefore, there is an imperative need to incorporate multimodal techniques, such as heart rate variability and functional Near-Infrared Spectroscopy, into future investigations. These methodologies would enable the real-time and objective assessment of physiological states and facilitate their systematic integration into the theoretical framework.

Thirdly, it is essential to conduct further investigations into the universality of the conclusions and their dynamics under different constraints. Most existing studies have been carried out in controlled laboratory settings, leading to a disconnect between the employed task paradigms and the highly dynamic, high-perceived exercise load decision-making environments encountered in real-world sports scenarios. This discrepancy raises concerns regarding the validity of the research findings. Furthermore, variations may occur in the participation and interaction patterns of the two systems under different task constraints, such as open versus closed skill activities and team versus individual projects. Existing theoretical frameworks are inadequate to comprehensively explain this dynamic adaptability in relation to task characteristics.

Fourth, theoretical verification methodologies are still underdeveloped, and there is a significant lack of computational models that can accurately quantify the interaction processes between the two systems. Most contemporary research primarily depends on behavioral metrics, such as reaction time and accuracy rate, or isolated neurophysiological indicators to indirectly infer the activities of the dual system. This methodology is deficient in rigorous mathematical models and data analysis techniques capable of accurately characterizing the dynamic trade-off relationship between the two systems. As a result, the investigation of propositions concerning the dual system remains largely theoretical.

In summary, although current research on sports decision-making based on two-system theory has led to significant progress, it encounters challenges concerning integration, accuracy, ecological validity, and methodology. These limitations highlight the imperative to develop an innovative theoretical framework that integrates the diverse dimensions of task constraints, physiological states, and experience levels, while clarifying their dynamic interactions. This pursuit is crucial not only for theoretical advancement but also as a necessary response to the complexities inherent in practical movement decision-making.

## 3 Theoretical framework construction

### 3.1 Frame core proposition

**Figure 1:**
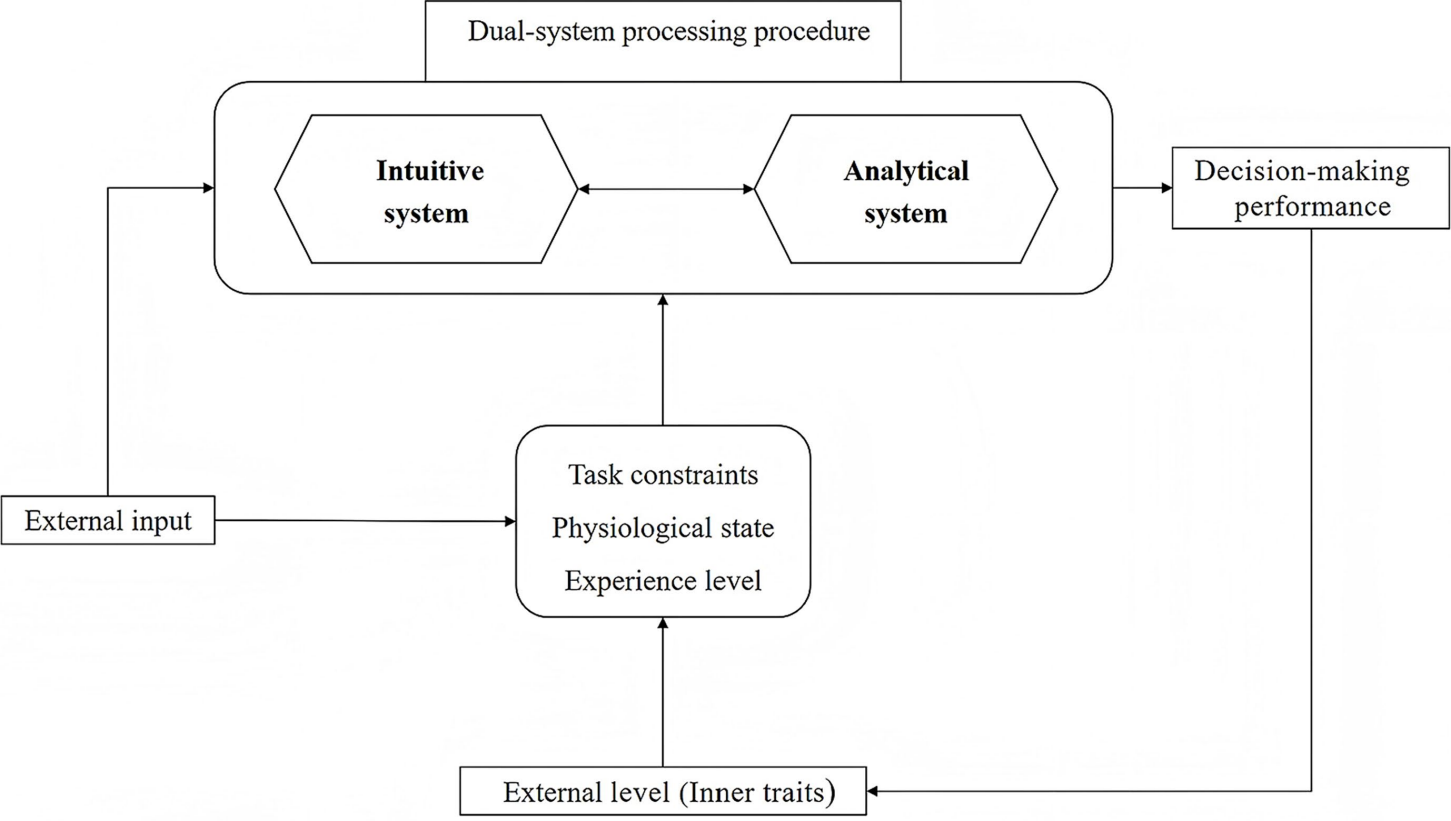
Schematic diagram of dual-system three dimensional dynamic theoretical framework for motor decision.

In this paper, based on the above theory combing and research limitations, a new three-dimensional dynamic theoretical framework is proposed (see Figure 1). The core proposition of this framework is that the dynamic trade-off and synergistic efficiency of two systems in exercise decision-making are not determined by a single factor, but are jointly regulated by three dimensions of external task constraints, individual physiological state and experience level and their interactions. This framework is proposed to go beyond the relatively static binary division of traditional two-system theory and provide a dynamic and situational interpretation perspective. In contrast to traditional research paradigms, fundamental advances have been made in clarifying that individual differences (e.g., immediate physiological state versus long-term experience) are key to different decision-making outcomes. Traditional isolated research perspectives are difficult to capture such nonlinear interaction effects, and this framework is proposed to treat decision-making as a dynamic system with multi-factor collaborative configuration, rather than a simple switch between two systems, thus providing a more practical theoretical tool for understanding decision-making behavior in real sports situations.

Meanwhile, the framework is built on three basic principles:

One principle is that of multidimensional interaction, which posits that task constraints, physiological states, and experience levels form a dynamic, integrated system. The specific pathways through which these elements interact are exemplified as follows: the dimension of task constraints, such as time pressure and task complexity, serves as external inputs that directly influence the engagement of the two systems. Elevated time pressure, in particular, substantially enhances dependence on intuitive system, while concurrently reducing the engagement of analytical system (Paras et al., 2015; Maltsev et al., 2023). The dimension of physiological state, encompassing factors such as fatigue and arousal, constitutes the neurodynamic foundation for decision-making processes. It modifies the equilibrium between the two systems by regulating the accessibility of cognitive resources. For instance, elevated levels of fatigue deplete cognitive resources and directly impair PFC function, compelling decision-makers to increasingly depend on intuitive system,even in challenging circumstances. Conversely, an optimal level of arousal facilitates the effective functioning of analytical system (Venkatraman and Wittenbraker, 2020). As a relatively stable intrinsic characteristic, the primary function of the experience level dimension is to modulate the operational mode of the previously mentioned dimensions and factors, as indicated by the dotted arrow in the figure. Expert athletes, possessing advanced structured knowledge representation and pattern recognition abilities, are able to utilize intuitive system processes with greater reliability in familiar situations. Concurrently, they can direct analytical system analytical processes toward novel and more complex challenges, thus facilitating an optimal allocation of cognitive resources (Paras et al., 2015). The nonlinear interaction of these three dimensions cannot be captured by traditional research paradigms. For example, novices may rely entirely on under-optimized intuitive system due to the weakened function of analytical system under fatigue, resulting in frequent errors, while experts can maintain stable performance with highly automated intuitive system under the same conditions.

The second principle is that of dynamic adaptability, which posits that optimal decision-making performance is not achieved through the absolute dominance of a single system. Instead, it relies on the dynamic and adaptive switching between two systems. Furthermore, this ability to adaptively switch is considered a trainable advanced cognitive skill. This principle is operationalized within the framework as follows: the dynamic interplay among the three dimensions—task constraints, physiological state, and experience level—collectively influences the modes of competition, substitution, or cooperation within the dual-system processing mechanism, ultimately manifesting as decision performance. Furthermore, the outcomes of decision performance reciprocally impact the physiological state and experiential accumulation of decision-makers through feedback loops. Consequently, a primary objective of the framework is to elucidate the specific patterns that govern dynamic adaptive switching across various combinations of dimensional contexts.

The third principle is measurability. The primary variables associated with the three dimensions outlined in this paper are both operational and quantifiable. This establishes a foundation for empirical research on the subsequent framework, enabling the use of multimodal techniques to concurrently collect behavioral, physiological, and neural data. Such an approach facilitates a comprehensive characterization of the cognitive neural processes through which these three dimensions collectively influence decision-making.

This framework prioritizes task constraints, physiological state, and experience level as its principal dimensions. In the interim, prior research has substantiated that these three dimensions systematically and directly influence the availability of cognitive resources as well as the operational mode of the dual-system (McClure and Bickel, 2014; Esmaeili Bijarsari, 2021; Amico and Schaefer, 2022; Baldacchino et al., 2022; Alister et al., 2024; Wang, 2025; Zucchelli et al., 2025). While acknowledging the substantial impact of cognitive states (e.g., attention) and personality traits, these factors have been intentionally omitted to maintain theoretical parsimony and to focus on the most direct and quantifiable factors that link the external environment, internal resources, and long-term adaptation. Future iterations of the model may explore the inclusion of these elements as moderating variables. It is upon this focused foundation that this framework seeks to transcend the previous paradigm of examining individual factors in isolation. It underscores the importance of accounting for the external task requirements encountered by decision-makers, alongside their physical condition and the cognitive resources developed through long-term training. This comprehensive approach aims to more accurately and thoroughly elucidate the complex nature of exercise decision-making.

### 3.2 Dimension connotation and function mechanism

#### 3.2.1 Task constraint dimensions

Task constraints refer to objective conditions and requirements set by the task itself in exercise decision-making. As external manipulable variables, task constraints directly determine the participation degree and action mechanism of intuitive system and analytical system by adjusting available cognitive resources and processing depth. This dimension mainly includes two core elements: time pressure and task complexity.

Time stress pertains to the psychological pressure individuals encounter when required to make decisions within constrained timeframes, influencing dual-system synergy by altering the allocation of cognitive resources (Raab, 2002). Under conditions of high time pressure, there is a marked tendency for the dominance of intuitive system to increase, leading individuals to depend more heavily on intuitive and heuristic strategies. Concurrently, this pressure significantly impedes the functioning of analytical system, thereby obstructing thorough analysis and rational computation(Yang et al., 2025). At the behavioral level, the velocity of decision-making is enhanced; however, this is concurrently associated with a reduction in accuracy and an alteration in risk tolerance (Raab, 2002). The results of empirical studies also support the above views: cognitive load experiments show that time pressure can enhance intuitive system and inhibit analytical system (Yang et al., 2025);ultimatum Game studies show that individuals are more likely to accept unfair offers under time pressure, reflecting impaired rational judgment (Wei et al., 2022);eye movement studies show that fixation time is shortened and strategies are simplified, indicating that information processing tends to be superficial (Wei et al., 2022). The neural mechanism research further shows that time stress can enhance the activation of emotion-related brain areas such as Amy and weaken the activity of cognitive control brain areas such as PFC (Cui et al., 2022),providing physiological evidence for the imbalance of dual systems under stress.

Task complexity, defined by the information, steps, and decisions involved (Ham, 2013), directly affects cognitive load. Tasks of high complexity require a greater allocation of cognitive resources, predominantly engaging analytical system for conscious regulation (Wei et al., 2022). In contrast, tasks of low complexity are typically processed automatically by intuitive system (Da Silva, 2023). As task complexity escalates, individuals are required to enhance their cognitive control to effectively suppress interference and sustain goal-directed behavior (Cui et al., 2022). Consequently, the interaction between the two systems transitions to a state where analytical system monitors and modifies the output of intuitive system (Bellini-Leite, 2017; Gómez Tabares, 2022). At the behavioral level, increased task complexity is typically associated with prolonged reaction times and elevated error rates, as well as a decline in decision quality attributable to cognitive overload (Sintchenko and Coiera, 2003; Jang et al., 2022; Zhou et al., 2022; Chen et al., 2023). Neuroimaging research indicates that tasks of low complexity predominantly engage brain regions associated with automatic processing, such as the sensorimotor cortex (Glass, 2019). In contrast, tasks of high complexity markedly increase activation and functional connectivity in brain regions involved in cognitive control and working memory, including the PFC (di Bello et al., 2024).

It is crucial to recognize, however, that the impact of task constraints on dual systems is not unequivocal. The inhibitory effect of high time pressure on analytical system may be attenuated in expert athletes (Zoudji et al., 2010; Guo and Wang, 2025),and the demand for analytical system resources in tasks of high complexity may fluctuate based on individual differences (Moreira et al., 2021). Therefore, task constraints dynamically affect the participation patterns of the two systems by regulating the activation levels and functional connections of key brain networks such as PFC, but their effects are also regulated by factors. Studying the dimension of task constraints in isolation is insufficient. To thoroughly comprehend the dynamic interaction mechanisms involved in decision-making within two-system motion, it is imperative to integrate this analysis with an investigation of the decision maker’s internal state.

#### 3.2.2 Physiological state dimension

Physiological states constitute the neurodynamic foundation of motor decision-making, with alterations in these states modulating the dynamic equilibrium between the two systems by influencing the efficacy of brain networks. This study concentrates on fatigue and arousal, two fundamental physiological factors that modulate the overall resources available for cognitive processing. They achieve this by altering the neurochemical environment and functional brain connectivity, thereby impacting the engagement of intuitive system and analytical system.

Fatigue depletes cognitive resources and impairs executive function, hindering analytical system. Neuroimaging reveals reduced dorsolateral PFC activation and weaker connectivity to the posterior parietal cortex during fatigue, diminishing analytical system’s regulatory capacity (Xie et al., 2023). Numerous empirical studies corroborate this phenomenon: financial analysts increasingly depend on heuristic decision-making associated with intuitive system following prolonged periods of work, which leads to a marked decrease in prediction accuracy (Hirshleifer et al., 2019). Similarly, surgeons tend to adopt more conservative approaches toward the conclusion of surgical schedules, indicative of a reduction in their analytical capabilities (Hirshleifer et al., 2019). Moreover, fatigue increases the predominance of intuitive system, thereby modifying individual risk tolerance and impairing feedback learning capabilities. Fatigue heightens ventromedial prefrontal cortex activity, indicating more emotion-driven decision-making (Jia et al., 2022). Effects of mental fatigue on risk preference and feedback processing in risk decision-making (Gantois et al., 2019; de Sousa Fortes et al., 2023). However, techniques such as transcranial direct current stimulation can effectively alleviate the negative effects of fatigue and improve the accuracy of decision-making, which also confirms the key role of PFC in fatigue decision-making and the plasticity of analytical system function (Qi et al., 2025).

As a critical physiological regulatory factor, the degree of arousal primarily facilitates the release of norepinephrine (Munn et al., 2021)via the ascending arousal system of the brainstem. This process dynamically modulates cortical neural activity, thereby influencing the decision-making process. According to the two-system theory, arousal levels influence the use of cognitive resources: low arousal favors intuitive decisions by intuitive system, increasing bias risk, while high arousal enhances analytical system’s analytical skills but can lead to cognitive overload. According to the Yerkes-Dodson law, simple tasks improve with high arousal, whereas complex tasks achieve optimal performance at medium arousal levels (Sörensen et al., 2022). Neurological studies have also shown that the Orbitofrontal Cortex and Dorsal Anterior Cingulate Cortex are involved in the interactive regulation of arousal states and decision-making in the body (Fujimoto et al., 2021),and fMRI data show that the strength of functional connections between the ascending system and cortex can directly shape cognitive state expression (Munn et al., 2021). Behavioral research has demonstrated that phased arousal can effectively mitigate decision bias and enhance the precision of evidence accumulation (de Gee et al., 2020). Additionally, elevated arousal levels have been shown to augment the functioning of analytical system, thereby encouraging decision-makers to adopt a more risk-averse approach (Alsharawy et al., 2021). Thus, arousal plays a dynamic balancing role in decision-making: intuitive system relies on higher arousal to achieve rapid response, while analytical system requires moderate arousal to maintain cognitive control, and its overall effect is modulated by task complexity and individual state (Jahedi et al., 2017).

Based on the above, fatigue mainly weakens the neural basis of analytical system, causing the decision to shift to intuitive system, and arousal adjusts the balance between the two systems in a nonlinear way. Therefore, the performance of exercise decisions depends not only on external task constraints, but also on the real-time impact of their internal physiological state, which also highlights the importance of monitoring and quantifying physiological states in real sports scenarios.

#### 3.2.3 Experience level dimension

The expert-novice paradigm in cognitive psychology examines how experts and novices differ in problem-solving, learning, and decision-making. Research indicates that experts, through extensive experience, can rapidly recognize patterns and break down tasks, primarily using intuitive system for swift decision-making. This intuition stems from the internalization of experience (Aven, 2018). Importantly, this expertise advantage is substantiated by empirical evidence rather than anecdotal accounts; a comprehensive three-level meta-analysis quantitatively reveals a substantial expert advantage in action anticipation, with a Hedges’ g of 1.15. This finding highlights the enhanced perceptual-cognitive skills that facilitate the effective functioning of the intuitive system in experts (Song et al., 2025b). Furthermore, an independent meta-analysis corroborates that this expertise advantage encompasses automatic prediction in visual motion representation, revealing a substantial effect size (Hedges’ g = 0.73) that is generalizable, consistent, and prevalent across various domains (Song et al., 2025a). Novices lack the experience for reliable intuition and must depend on analytical system analysis, which is rule-based and easily swayed by superficial details, leading to slow and inflexible decision-making (Venkatraman and Wittenbraker, 2020). The accumulation of experience facilitates the transition from novice to expert, fundamentally altering the operational dynamics of dual-process systems. Significantly, this developmental trajectory is active rather than passive. A distinct meta-analysis corroborates that visual anticipation skills can be markedly enhanced through targeted Temporal Occlusion Training, thereby illustrating the plasticity of the perceptual-cognitive processes involved in expert decision-making (Müller et al., 2024). In situations where experience is lacking, individuals predominantly depend on the logical reasoning and information evaluation capabilities of analytical system to compensate for the unreliability of their intuition (Aven, 2018). As experience accumulates over time, a deeper understanding of the domain enhances the reliability and efficiency of intuitive judgments made by intuitive system (Aven, 2018), and the role of pattern recognition, automatic reaction and emotional intuition in decision-making is significantly enhanced, reducing the dependence on conscious analysis of analytical system. Ultimately, leveraging their experience, experts can enhance the cooperative interaction between intuitive system and analytical system during complex tasks, thereby facilitating improved decision-making.

Experience-driven behavioral changes are linked to neural mechanisms, primarily seen in neuroplasticity, optimized cognitive control, and intuitive processing. Research indicates that extended specialized training strengthens neural connections and boosts information processing speed and accuracy (Purcell et al., 2020). In terms of cognitive control, experienced individuals are able to use PFC more efficiently, effectively suppress extraneous interference and focus on key information. As experience grows, intuitive judgment becomes reliable, linked to adaptive changes in brain activity patterns like those in the Amy and BG. These regions are capable of developing more refined neural representations, which enhance the precision of pattern recognition and the accuracy of outcome prediction (Cui et al., 2022). Moreover, experiential factors may modulate the activity patterns of the Default Mode Network, which is predominantly associated with resting self-referential cognition and situational simulation. Alterations in these patterns may subsequently affect individuals’ perceptions and expectations regarding their own behavior as well as that of others (Cui et al., 2022).

In conclusion, the level of experience significantly influences the dynamic interaction patterns between the two cognitive systems through neural remodeling. Novices predominantly depend on analytical system for explicit analytical processes, whereas experts primarily utilize the capabilities of intuitive system, with the ability to flexibly engage analytical system for monitoring and corrective purposes as necessitated by the task context. The enhancement in neural efficiency and the automation of processing patterns offer neurobiological insights into how expert athletes make optimal decisions in complex, dynamic environments. Consequently, it is challenging to derive comprehensive and universally applicable conclusions without considering empirical evidence in the discourse on sports decision-making research.

### 3.3 Framework operationalization and validation path

In order to enable the theoretical framework to accept empirical testing and guide subsequent data collection and analysis, this section aims to propose operational ideas at the theoretical level and provide top-level design guidance for subsequent research. This paper advocates adopting.

The dual-path research strategy of combining laboratory and real sports situations verifies the framework proposition systematically and in stages by considering the high internal validity of laboratory environment and the high ecological validity of real situation.

Based on the stage of the laboratory study, core variables were manipulated by precise experimental design to determine causality. Task constraint dimensions can be precisely manipulated through computerized task paradigms, such as setting decision windows of different lengths to simulate time pressure, or manipulating task complexity by adjusting the number and conflict of information options. The physiological state dimension needs to be induced and measured by standardized procedures, such as inducing controllable fatigue state through quantitative cognitive or physical load exercise, and collecting subjective self-evaluation and objective physiological indicators simultaneously. The dimension of experience level was based on objective criteria such as training years and sports grades, strictly recruiting and distinguishing experts from novices. The core of this stage is to use high-precision equipment such as high-density EEG and eye tracker to deeply explore the cognitive neural mechanisms of the two systems. Then, based on the real sports situation research stage, we need to test the ecological validity of the framework proposition on the sports training or competition site. Task constraint dimensions can be obtained through live video recording and post-event expert annotation analysis, such as the remaining time of an attack, the number of pass options, etc. The physiological state dimension indicators rely on wireless sensing devices such as portable multi-channel instruments and EEG devices for synchronous collection in natural situations. The experience level grouping is consistent with the experimental phase. This phase focuses on verifying whether laboratory findings can be reproduced in real and dynamic sports environments and completing the final test of the external validity of the theoretical framework.

To ensure the consistency of the core dependent variable measurement across the two phases, it is imperative to consider the level of data integration and validation. Subsequently, statistical methodologies, including the Multilevel Linear Model, were employed to examine the combined effects of three dimensions—task constraints, physiological state, and experience level—along with their interaction terms, on decision-making behavior and neural activity. This analysis serves to elucidate the central aspects of the theoretical framework. The hypotheses were quantitatively tested.

## 4 Research prospects and implications

A central feature of the theoretical framework developed in this paper is its verifiability (refer to Section 3.3). This framework not only offers a theoretical explanation but also elucidates the operational measurement pathways across three dimensions: task constraint, physiological state, and experience level, thereby establishing a robust foundation for empirical testing. Nonetheless, several methodological and technical challenges must be addressed to translate this theoretical framework into empirically verifiable studies and, ultimately, practical applications. In light of these challenges, this chapter will concentrate on the verification and application of the framework, examining the primary research directions and feasible approaches for future exploration from the perspectives of methodology and AI technology.

### 4.1 Methodological perspectives: multimodal measurement and delphi

Empirical investigations of theoretical frameworks encounter a range of methodological challenges. This section seeks to examine three categories of these challenges and to offer insights into future research trajectories and critical technological pathways to address them.

One critical consideration is the balance between ecological validity and measurement accuracy. While the laboratory environment allows for precise control, its simplified paradigms often fail to capture the full complexity and uncertainty inherent in real-world motion. Conversely, real-world motion scenarios ensure ecological validity but present challenges such as significant signal interference and difficulties in controlling variables. Building on the aforementioned considerations, future research should prioritize the development and implementation of lightweight, highly portable, and multi-scene adaptive wireless sensing technologies. These advancements aim to facilitate the high-quality collection of neurophysiological data while minimizing disruptions to athletes’ regular training and competitive activities.

The second aspect involves the synchronization and integration of multimodal data. Various data types, including behavioral, physiological, and neural data, exhibit distinct temporal resolutions and physical characteristics. Achieving synchronization among these diverse data forms is fundamental for subsequent analytical processes. Future research must urgently focus on developing a technical process that integrates hardware synchronous triggering with software post-event alignment. For instance, employing a unified wireless signal transmitter to disseminate timestamps to all acquisition devices could facilitate this integration. Additionally, the behavior time points captured by high-speed cameras could be utilized for subsequent fine-tuning adjustments, thereby enabling the construction of a multimodal database aligned with a unified timeline.

The third aspect involves the standardization and systematization of measurement indicators. The primary objective of subsequent research is to develop a core index system for the three-dimensional dynamic theoretical framework using the Delphi method. Concurrently, it is essential to establish an expert panel comprising specialists in cognitive neuroscience and sports psychology. Through multiple rounds of anonymous structured questionnaires, the panel should aim to reach a consensus on the importance, sensitivity, and feasibility of various candidate indices. Ultimately, this process should yield a set of efficient, reliable, and widely recognized core indices. This endeavor will provide standardized measurement tools and a methodological foundation for the empirical investigation of the framework.

### 4.2 Application prospects of artificial intelligence technology

AI, as a cutting-edge technology, provides innovative method support and tool support for the research and practice of motor decision-making double system theory. It can not only extract knowledge and rules from complex data with the help of machine learning technology, but also endow robots with the ability of perception, learning and decision-making through intelligent technology, which also provides a new analytical perspective and research path for in-depth understanding of the interaction mechanism of dual systems.

According to intuitive system, AI leverages three types of path enablement: processing extensive game videos and motion data using machine learning and deep learning to uncover hidden rules like opponent tactics and individual actions, enabling behavior prediction and real-time decision support (Antalamarad and Upadhye, 2024). Secondly, utilizing multi-scheme evaluation capabilities, offer optimal decision-making recommendations for athletes engaged in high-speed confrontational sports, such as basketball and football (Liu, 2020; Li, 2025). Third, the incorporation of virtual reality and augmented reality technologies to develop highly simulated training scenarios, in conjunction with an AI-driven personalized feedback mechanism, significantly enhances athletes’ subconscious reaction capabilities (Qi et al., 2024). According to analytical system, the supportive function of artificial intelligence is of equal importance. At the data processing stage, AI facilitates the integration of multi-dimensional information, including athletes’ physiological indicators, competition footage, and intelligence on opponents. This integration enables the extraction of tactical patterns through data mining, thereby establishing a foundation for scientific decision-making. In the realm of strategic optimization, a digital twin model of complex game scenarios is developed to precisely assess the efficacy of various tactical combinations. This includes predicting opponents’ responses through simulated lineup matching, ultimately generating optimal tactical solutions (Palacios Zumba et al., 2024; Wbaid et al., 2025). Simultaneously, it becomes feasible to implement personalized training tailored to individual characteristics. AI dynamically adjusts training content and intensity threshold by analyzing athletes ‘technical shortcomings and advantages, so as to realize accurate improvement of skills (Chen, 2024; Raveena and Rathod, 2024).

The application of artificial intelligence in specific sports scenarios further demonstrates its feasibility. In the context of basketball, AI optimizes shooting techniques by analyzing the dynamics of shooting characteristics and predicts opponent defensive formations, thereby facilitating the strategic planning of offensive routes (Liu, 2020). In football sports, player movement thermal diagrams and pass success rate models are utilized to reconstruct tactical spatial layouts. Simultaneously, these tools are employed to predict the opponent’s offensive intentions, thereby enhancing defensive decision-making (Antalamarad and Upadhye, 2024). In track and field events, gait analysis and optimization of starting angles are employed to enhance athletic performance, while the integration of a physiological index warning system is utilized to mitigate the risk of injury (Aarons et al., 2023). At the strategic decision-making level in coaching, artificial intelligence can facilitate the evaluation of substitution strategies and the adjustment of tactics under time constraints, thereby significantly enhancing coaches’ real-time command capabilities (Aarons et al., 2023).

Building on the previous discussion, AI has made notable strides in motor decision theory. Current studies show that deep learning can simulate decision-making and explore its real-time use in complex scenarios. However, most research focuses on offline analysis. More work is needed on adaptability, interpretability, and human-computer collaboration in real environments. Future research should aim to create intelligent decision systems that integrate multi-modal data, brain-like computing, and adaptive learning, advancing AI from theory to practical decision support in dynamic settings.

## 5 Conclusion

### 5.1 Theoretical summary

Building upon the static and binary constraints inherent in dual-system theory within the context of motor decision-making research, this paper introduces a novel three-dimensional theoretical framework that integrates task constraints, physiological states, and experience levels (see Figure 1). The primary advancement of this framework is its departure from the traditional single-variable analysis approach, by incorporating three dimensions into a unified explanatory mechanism for the first time. This framework systematically elucidates how it modulates the participatory weight and cooperative dynamics of intuitive system and analytical system through a nonlinear interactive process, ultimately influencing decision-making performance.

The primary theoretical contributions of this framework are threefold, addressing longstanding criticisms and gaps in the literature. First, it directly addresses the prevalent criticism of the static nature of dual-system theory by reconceptualizing it as a dynamic, context-dependent model in which system dominance is continuously influenced by the interplay of task constraints, physiological states, and experience levels. Second, it introduces an innovative multi-dimensional interaction perspective, moving beyond the isolated factor analyses that have characterized previous research, to provide a comprehensive account of how external constraints and internal states interactively shape decision-making processes. Third, it establishes empirically testable pathways through the explicit integration of multimodal measurement and computational modeling, thereby bridging the gap between high-level theoretical description and concrete, quantitative prediction. This approach shifts the field from debating the existence of two systems to investigating the precise conditions that govern their interaction.

The theoretical innovation and core values of the framework focus on the following three points:

Initially, this approach represents a paradigm shift from static descriptions to the dynamic diagnosis of mechanisms. The framework moves beyond a singular perspective, offering a comprehensive analytical tool. By collaboratively analyzing external task scenarios (such as time pressure and complexity), individual real-time states (including fatigue and arousal), and long-term experiential traits, it allows for the precise identification of the underlying mechanisms contributing to decision errors. For instance, a missed pass can be attributed to a confluence of stressful conditions, physical fatigue, and inexperience, which collectively impair analytical system functioning and weaken intuitive system.

Secondly, it offers a comprehensive mechanistic explanation for the variability in athletes’ performance. The fluctuation in athletes’ states fundamentally represents a distinct characteristic of the equilibrium point migration within a dual-system framework, driven by three-dimensional dynamic coupling. This framework highlights the importance of continuously monitoring decision evaluation over time, illustrating how athletes shift from using analytical system for complex analysis at the start of a competition (when fatigue is low and arousal is optimal) to relying on intuitive system as fatigue increases and arousal becomes disordered, thereby explaining performance fluctuations.

Third, this study redefines the concept of expert advantage and informs the direction of precise training interventions. The findings suggest that expert advantage is not solely attributed to the rapid automation of intuitive system processes, but also to the metacognitive ability to efficiently and flexibly switch between the two systems through neural remodeling. At the application level, the framework translates performance issues into targeted intervention strategies: when physiological indicators remain stable under high-pressure conditions, it is advisable to enhance intuitive training; when attention is compromised due to inadequate arousal, psychological adjustment should be implemented; and when there is difficulty in comprehending complex tactics due to a lack of experience, emphasis should be placed on tactical cognitive training.

Building upon the aforementioned analysis, this framework not only synthesizes and enhances prior research but also offers a conceptual foundation and methodological guidance for the paradigm shift from static separation to dynamic integration in motor decision research. This advancement holds significant theoretical value and potential for practical application.

### 5.2 Study limitations

The theoretical framework in this paper offers a fresh perspective on the interaction mechanism in motor decision-making. However, it relies heavily on logical deduction and existing literature, lacking strong empirical support, highlighting the need for future research to focus on empirical validation.

From a theoretical perspective, it is crucial to investigate the interaction effects among the three dimensions through empirical research. This includes examining how varying intensities of task constraints influence decision-making by modulating physiological states, and how this modulation effect differs based on the athletes’ levels of experience. Building on the proposed methodology, this paper introduces a multi-modal measurement approach; however, challenges persist in synchronizing and analyzing physiological and neural data. Furthermore, the applicability of this framework requires systematic validation across various sports disciplines. Current research predominantly emphasizes team ball sports, with a notable deficiency in studies pertaining to winter sports and emerging electronic sports. Finally, the framework’s emphasis on task constraints, physiological states, and experience levels, while advantageous in terms of parsimony and focus, also establishes a boundary condition. Influential factors such as transient cognitive states (e.g., attention fluctuations) and stable personality traits were excluded from the present modeling effort. Although this exclusion facilitates a clearer examination of the core dimensions, it is acknowledged that these omitted variables may contribute additional variance to decision-making performance. Future research should prioritize the development of methodologies to integrate these elements, potentially as moderating variables, to progress toward a more comprehensive model of athletic decision-making. Considering the level of application transformation, numerous complex challenges must be addressed to effectively translate the theoretical framework into a decision-making system that can be integrated into real-world training competition environments and future talent selection processes. Consequently, advancing sports decision-making research from theoretical elucidation to practical empowerment necessitates completing the closed-loop process from theoretical construction through empirical testing to practical application.

### 5.3 Future research direction

To ensure that the theoretical framework proposed in this paper receives empirical validation and demonstrates its practical applicability, it is essential to develop a standardized measurement system for the methodology. Subsequent research should focus on constructing a core index system tailored to sports decision-making research by employing expert consensus methods, such as the Delphi method. This process involves establishing key observational variables for each dimension, along with their operational definitions, thereby providing standardized measurement tools for empirical investigation.

Simultaneously, grounded in the theoretical framework, future empirical investigations should adopt a systematic methodology. Initially, it is imperative to integrate a multi-factor experimental design to examine the three-dimensional interaction effects of task constraints, physiological state, and experience level. This approach should emphasize the combined influence of various dimensional interactions on decision-making processes. Secondly, we can conduct an in-depth investigation into the neural mechanisms underpinning behavioral experiments and develop a pathway model that links physiological states to brain activity and subsequent decision-making behavior. This can be achieved through the utilization of multimodal neurophysiological indicators, thereby elucidating the neural foundations influenced by varying levels of experience. Thirdly, following extensive empirical research, it is imperative to advance computational modeling studies to quantify the dynamic principles governing the variation of dual-system weight in relation to three-dimensional degrees. This should be achieved by integrating behavioral and neural data to complete the theoretical-to-practical feedback loop. Consequently, this approach will facilitate the development of ecological application schemes grounded in wearable technology and artificial intelligence, thereby transforming theoretical insights into quantitative tools for guiding training practices and optimizing talent selection. Ultimately, this will enable the realization of the core value of translating theoretical empowerment into practical applications in sports.

## Data Availability

The original contributions presented in the study are included in the article/supplementary material, further inquiries can be directed to the corresponding author.
